# Expected values for pedometer-determined physical activity in older populations

**DOI:** 10.1186/1479-5868-6-59

**Published:** 2009-08-25

**Authors:** Catrine Tudor-Locke, Teresa L Hart, Tracy L Washington

**Affiliations:** 1 Walking Behavior Laboratory, Pennington Biomedical Research Center, Baton Rouge, LA 70808, USA; 2 Department of Exercise and Wellness, Arizona State University, Mesa, AZ 85212, USA

## Abstract

The purpose of this review is to update expected values for pedometer-determined physical activity in free-living healthy older populations. A search of the literature published since 2001 began with a keyword (pedometer, "step counter," "step activity monitor" or "accelerometer AND steps/day") search of PubMed, Cumulative Index to Nursing & Allied Health Literature (CINAHL), SportDiscus, and PsychInfo. An iterative process was then undertaken to abstract and verify studies of pedometer-determined physical activity (captured in terms of steps taken; distance only was not accepted) in free-living adult populations described as ≥ 50 years of age (studies that included samples which spanned this threshold were not included unless they provided at least some appropriately age-stratified data) and not specifically recruited based on any chronic disease or disability. We identified 28 studies representing at least 1,343 males and 3,098 females ranging in age from 50–94 years. Eighteen (or 64%) of the studies clearly identified using a Yamax pedometer model. Monitoring frames ranged from 3 days to 1 year; the modal length of time was 7 days (17 studies, or 61%). Mean pedometer-determined physical activity ranged from 2,015 steps/day to 8,938 steps/day. In those studies reporting such data, consistent patterns emerged: males generally took more steps/day than similarly aged females, steps/day decreased across study-specific age groupings, and BMI-defined normal weight individuals took more steps/day than overweight/obese older adults. The range of 2,000–9,000 steps/day likely reflects the true variability of physical activity behaviors in older populations. More explicit patterns, for example sex- and age-specific relationships, remain to be informed by future research endeavors.

## Introduction

The recently released U.S. Physical Activity Guidelines http://www.health.gov/paguidelines were informed by an advisory committee report that concluded that, for older adults, in addition to the well-know health benefits of a physically active lifestyle, "strong evidence indicates that being physically active is associated with higher levels of functional health, a lower risk of falling, and better cognitive functioning"[1]. Although we have a collective understanding that physical activity declines with age [2], we know little about the actual physical activity patterns of older adults. Accurate and precise quantification of physical activity behaviors is elemental to epidemiologists, physiologists, and behavioral scientists, as well as clinicians engaged in efforts to extend quality years of life and ultimately compress morbidity. In addition, researchers and practitioners must be prepared with practicable and appropriate assessment tools, including becoming competent in the interpretation of their data, if they are to successfully address measurement challenges related to surveillance, screening, intervention, and program evaluation.

Motion sensor technology has evolved quickly in recent years, producing a wide variety of commercially available body worn instrumentation, including accelerometers and pedometers, capable of objectively detecting physical activity behaviors. Although accelerometers can offer minute-by-minute estimates of physical activity volume and intensity, their relatively high costs, data management demands, and tandem requisites for personnel time and technical expertise stand as a barrier to widespread implementation outside formal research studies. In contrast, simple and affordable pedometers are generally considered more practical for individual, clinical, and population level applications. In addition, pedometer outputs correlate highly with that of different accelerometers [3]. Although pedometers are not designed to directly detect physical activity intensity, they do provide a feasible and practical means of capturing daily physical activity volume (especially walking) expressed as a summary output of steps/day. Walking for exercise increases with age and it is also a fundamental behavior in daily life activities [4]. An indicator of volume is likely sufficient when studying older populations, who do not typically engage in high levels of physical activity intensity. Based on accelerometer data, Troiano et al. [5] reported that 50–59 year olds average 1.1 minutes daily in vigorous intensity activities, and 60–69 years olds and those over 70 years of age, respectively average 0.4 and 0.1 minutes in vigorous activity daily.

Pedometers are considered analogous to computer hardware (e.g., keyboards, monitors, disks, etc.); without the matching software (e.g., expected values, etc.) they are of limited utility [6]. Expected values are normative or benchmark values necessary for interpreting change and comparison purposes [7]. In 2001, we compiled expected values of free-living pedometer-determined physical activity in adults [8]. At that time (and based on only 4 studies [9-12]) we concluded that healthy older adults (≥ 50 years without chronic disease) accumulated 6,000–8,500 steps/day. Since then, pedometry as a scholarly and applied practice has burgeoned and the related scientific literature is again ripe for a comparative examination. Therefore, the purpose of this review is to present expected values for pedometer-determined physical activity published since the original review paper, pertinent to free-living healthy older populations.

## Methods for Literature Review

A search of the literature (current and verified as of January 05, 2009) began with a keyword (pedometer, "step counter," "step activity monitor" or "accelerometer AND steps/day") search of PubMed, Cumulative Index to Nursing & Allied Health Literature (CINAHL), SportDiscus, and PsychInfo. A publication date limit was set from 2001, coinciding with the year of publication of the original review [8]. All authors then participated in an iterative process of identifying and verifying studies of free-living pedometer-determined physical activity (captured in terms of steps taken; distance only was not accepted) in older adult populations described as ≥ 50 years of age (studies that included samples which spanned this threshold were not included unless they provided at least some appropriately age-stratified data) and not specifically recruited based on any chronic disease or disability. We excluded one study that presented pedometer data only in a figure form that required interpretation to extract data [13]; however, we decided to include a study by Moreau et al. [14] that appeared to report rounded values for steps/day rather than exact numbers. We included the single report with the largest sample size [15] reporting steps/day from the Nakanojo Study papers that used pedometers to assess physical activity in older adults. We also excluded two other papers [16,17] after confirming with authors (Strath and Swartz, personal communication) their overlap with other papers from the same group. Finally, since the explicit focus of this review was on healthy older populations we also excluded two free-living studies [18,19] and a case study [20] of older adults living in assisted living facilities. The majority of these residents walk at speeds less than 0.6 meters/second [21], a speed which produces lower gravitational forces than are typically detected by research-quality pedometers [19,22,23].

Once the studies were assembled, we collated them according to the original reference, country where study was conducted, sample descriptions (including sex, age descriptors as available, and verbatim additional descriptions of the sample, if provided), verbatim descriptions of pedometer brands used, monitoring frame (i.e., number of days worn), whether the instrument was sealed or unsealed (explicitly stated or implicit from requiring participants to self-record data) to the participant, and mean (or median if mean not reported) and standard deviation or standard error of steps/day for the total sample and/or stratified groups, as available. We also looked for any data treatment descriptions including identifying extreme values or addressing missing data. In the case of intervention or other longitudinal-type studies, only the baseline data were considered. Although in previous reviews [24,25], an overall median steps/day was imputed considering those studies originally reporting mean values, we determined that it was overly reductionistic to attempt to represent all older adults by a single value for pedometer-determined physical activity. Instead we offer the studies' data as published and only capture their range as an indicator of distribution.

## Findings

We identified 28 unique studies of pedometer-determined free-living physical activity in healthy older adult populations representing at least 1,343 males and 3,098 females (another 568 older adults were not identified by sex in the original studies [26-29]) ranging in age from 50–94 years. All are assembled by year of publication [see Additional file 1]. Inconsistencies in presentation across table columns reflect underlying inconsistencies in reporting across studies. Eighteen studies (64%) were conducted in the United States, 5 in Japan (18%), 2 in Australia (7%), and 1 each in Thailand and Belgium. A final study was a secondary analysis of data assembled from a number of different countries. Eighteen (or 64%) of the studies clearly identified using a Yamax pedometer model (i.e., Yamasa, Yamax, Digi-walker). Monitoring frames (where reported) ranged from 3 days to 1 year; the modal length of time was 7 days (17 studies, or 61%). Only 5 studies (15%) indicated that they used a sealed pedometer (1 study unsealed the pedometer one week, and sealed it the next). Mean pedometer-determined physical activity ranged from a low of 2,015 steps/day (in one sample of 85+ year olds) [30] to a high of 8,938 steps/day in a sample described as normal weight males, aged 51–88 years [31].

Four studies offered pedometer data by sex [31-34]. In all but one case (males vs. females 50–59.9 years of age [34]) males took more steps/day than females, ranging from a low difference of 497 steps/day [33] to a high of 1,450 steps/day [32] for similarly aged groups. A clear decline in steps/day within study-specific age groupings was consistently apparent in the four studies that provided age-stratified pedometer data [27,30,31,34]. Two studies examined pedometer data by BMI-defined weight status categories [31,35]; individuals classified as normal weight (BMI ≤ 25 kg/m^2^) took between 1,659 [31] and 4,060 steps/day [35] more than individuals classified as overweight/obese. Three studies [28,36,37] also provided at least some pedometer data stratified by race/ethnicity. In each study, samples identified as White or Caucasian took more steps/day than other race/ethnic groupings considered.

## Conclusion

This review represents an extension of our earlier efforts to inform expected values for free-living pedometer-determined physical activity, previously focused on general adult populations [8] and youth (including children and adolescents) [25]. Older adults embody a special population that can derive additional benefits from a physically active lifestyle [1]. The data assembled here, and the amalgamation of expected values ultimately distilled, are useful to clinicians and researchers alike. As previously stated, accurate quantification of physical activity behaviors is an important requisite for surveillance, screening, intervention and program evaluation, as well as more basic research questions.

In our first attempt to assemble expected values for different populations [8], we concluded that healthy older adults take between 6,000–8,500 steps/day. This earlier conclusion was based on only four studies with limited sample sizes. We acknowledged at that time that expected values should be refined as more pedometer studies emerged. Although we did impute overall median values for other similar reviews (considering underlying studies reporting original mean values) [25], we believed that, in the case of older adults as a broadly defined population, a single value for steps/day fails to capture the fact that older adults range in ability and habit with age as well as other factors. In recognition of this phenomenon, we also conclude that the range of 2,000–9,000 steps/day more likely reflects the true variability of physical activity behaviors in healthy older populations. We believe that is it prudent to designate rounded anchors for the ranges of such expected values (to not overstate precision), and the use of 1,000 steps/day increment is rationale [31]. One thousand steps are approximately equivalent to 10 minutes of brisk walking in healthy adults [38].

Pedometer-determined physical activity has been shown to be inversely related to age [39]. In our previous review of pedometer-determined physical activity for youth [25], we were able to construct a graph of sex- and age-specific expected values from age 6 to 18 years. Herein we observed a clear decline in steps/day in those studies reporting age-stratified data in older adults [27,30,31,34], however, the study-specific age groupings were broad and ultimately were not comparable between studies precluding an opportunity to draw more explicit conclusions about expected values for different ages of older adults. In order to extend and complete the step/day curve that has been initiated in young populations [25], researchers should continue to provide both sex- and age-specific data to the extent that it is possible in their original publications.

Most studies of free-living pedometer-determined older adult physical activity have been conducted in the United States, but none of the assembled studies can be considered nationally representative. The most frequently used pedometer was different models of the Yamax brand, manufactured in Japan and available through a variety of distributors around the world. In a review of literature focused on youth pedometer studies [40], we found that 30 of 34 (88%) studies identified used the Yamax pedometer. The Yamax pedometer is considered to be a criterion pedometer, against which others may be compared [41]. However, its reduced sensitivity to low force ambulation is well known [42,43], and there are concerns that these pedometers miss steps in gait-impaired elderly [23]. Other pedometers and accelerometers offer reduced sensitivity thresholds, capable of detecting such low force movements [19,44,45]. However, the inevitable sensitivity-specificity trade-off can result in the over-detection of erroneous steps [44,45] and complicates comparison and amalgamation of data between studies, the central focus of this review. Further, instrumentation that is greatly discrepant from established research quality pedometers would require its own normative data for interpretation purposes. Even if low force ambulation is missed, however, the steps that are detected are considered to be above 0.35 G (i.e., the manufacturer-reported force threshold for the Yamax pedometer) [46], offering an unyielding standard that ultimately simplifies comparisons of the same measurement across populations and studies. Although it remains plausible that at least some age-related decline in steps/day is complicated by slower gaits with advanced age and disability, the pedometer-determined pattern displayed herein is congruent with what we know from other assessments of physical activity in these populations, including doubly labeled water [47,48]. Ultimately, the choice of the instrument used is a function of the research question, participant burden, and resources available to the researcher or practitioner.

As noted above, reported monitoring frames ranged from 3 days to 1 year although the most frequently reported length of time was 7 days. Three days is considered sufficient to capture habitual pedometer-determined physical activity (defined as the mean of 7 days) in healthy adult populations [49,50]. Sedentary populations with chronic illnesses appear to require even fewer days to obtain a stable estimate of habitual physical activity [51,52]. Likewise, Rowe et al. [53] studied older adults (over 60 years of age) and reported that between-day reliability was high (ICC = .90 for Yamax steps and .87 for ActiGraph steps) and that likely only 2 days are sufficient to obtain a stable estimate. We conservatively refrained from conducting any analyses to examine the relationship between monitoring frame and step count since it would likely be confounded by study differences in sample characteristics, sample sizes, instrumentation, assessment protocols, etc.

Relatively few studies used sealed pedometers to blind participants to their step counts. Since research participants can typically view unsealed pedometer data displays and observe how many steps they are accumulating, there is a concern that certain individuals may set out to accrue more steps, either because they are trying to please the researcher or because they are naturally motivated to achieve. Marshall et al. [54] examined this question in older adults by comparing data collected under unsealed and subsequently sealed conditions. Participants accumulated 400 more steps/day (*p *= 0.02) in the unsealed condition. The authors concluded that although the difference in steps/day was statistically significant, it might not be considered clinically significant. Put in context, this magnitude of difference attributed to reactivity is substantially less than what has come to be expected of successful pedometer-based interventions [55,56].

There was largely no reporting whatsoever of data treatment regarding missing data or extreme values. King et al. [57] indicated that "when a participant returned her activity diary with missing data, she was asked to start a new diary and wear the pedometer for another week." Fukukawa et al. [58] indicated that "to estimate the participants' usual walking activity, we discarded the maximum and minimum daily records from the entire data." Tudor-Locke et al. [34] wrote that "Data for any single day indicating < 1,000 steps were removed and values > 30,000 steps on any single day were truncated (i.e., replaced with 30,000 steps)." No other study described any other treatment for missing data or extreme values.

We located only two longitudinal studies of older adult pedometer-determined physical activity at this time [34,58]. Fukukawa et al. [58] collected two waves of data from 314 older adults (65–79 years of age) separated by two years. Values increased 1,483 steps/day over time. The participants were not part of an intervention and the authors did not offer any explanation for the observed increased. Another sample that included older adults (e.g., 50–71 years of age) was followed before and one year after they moved residences within Perth, Australia [34]. An overall small decrease in steps/day was evident with mean values ranging from -100 steps in 50–59.9 year old males and -776 steps in 60+ year old males to -407 in 50–59.9 year old females and -408 steps in 60+ year old females. Spearman rank order correlations were computed to assess relative stability of behavior over time. Males and females ranging in age from 50–59.9 years were moderately stable over one year (*r *= 0.533 and 0.645, respectively). Males aged 60+ years were also moderately stable (*r *= 0.542), however, the oldest females were less stable over time (*r *= 0.304).

It is important to stress that the expected values assembled herein do not envisage what older adults "should" be taking (i.e., an "indicator," "cut point," or "threshold" value). A recent analysis identified preliminary steps/day cut points for adults that best discriminated between BMI-defined normal weight and overweight/obesity [31]. Simultaneous consideration of a number of different indices produced estimates of 11,000 steps/day for males age 51–88 years (based on n = 220), 10,000 steps/day for females aged 50–59.9 years (n = 366), and 8,000 for females aged 60–94 years (n = 214). These values are obviously much higher than most of the expected values collected herein [see Additional file 1], and reflect the fact that expected values are frequently lower than criterion-based values [31]. Rowe et al. [53] conducted Receiver Operator Curve analysis to identify steps/day associated with achieving 30 minutes of continuous moderate-to-vigorous physical activity (typically associated with public health recommendations for physical activity) by older adults (mean age 74 years). An estimate of 7,000 to 8,000 steps/day was determined. It is important to emphasize that the first study concerned itself with steps associated with a healthy BMI and the second one focused on time in moderate-to-vigorous physical activity; these are distinct measurement criteria used to evaluate "how many steps are enough?" There are no other studies at this time that have attempted to address this question for older adults in particular.

In summary, expected values for pedometer-determined physical activity are necessary to enable research and program planning and aid in interpretation of similar data. The literature focused on older adults has expanded since an initial review published in 2001 [8], presenting a timely opportunity to amass comparable parameters and refine expected values. We located 28 studies of pedometer-determined free-living physical activity in healthy older adults ranging in age from 50–94 years. Steps/day ranged from 2,000–9,000 steps/day, effectively capturing the diversity of age. There is limited data portraying sex- and age-specific values for steps/day, however. These are necessary if we are to complete a fully evolved "life step curve," expanding on earlier versions of a youth step curve [25]. This shall hopefully be addressed as more pedometer-based research is accumulated and expected values continue to be cultivated.

## Competing interests

The authors declare that they have no competing interests.

## Authors' contributions

CTL conceived the review and lead its implementation and writing; TLW led the electronic search and TLA administered the database. All authors took part in identifying and abstracting articles and editing the final version. All authors read and approved the final manuscript.

## Supplementary Material

Additional file 1 **Table 1. Expected values for pedometer-determined physical activity in healthy older adults**. Compiled pedometer studies abstracted from the scientific literature. Click here for file
