# Correction: Expected values for pedometer-determined physical activity in older populations

**DOI:** 10.1186/1479-5868-6-65

**Published:** 2009-10-09

**Authors:** Catrine Tudor-Locke, Teresa L Hart, Tracy L Washington

**Affiliations:** 1 Walking Behavior Laboratory, Pennington Biomedical Research Center, Baton Rouge, LA 70808, USA; 2 Department of Exercise and Wellness, Arizona State University, Mesa, AZ 85212, USA

## Abstract

Correction to Tudor-Locke C, Hart TL, Washington TL: **Expected values for pedometer-determined physical activity in older populations**. *International Journal of Behavioral Nutrition and Physical Activity *2009, **6:**59

## Correction

After publication of this work [1], we noted that some of the reference numbers in Additional file 1 were incorrect [2-29]. Here we provide the correct table and new reference list.

## Supplementary Material

Additional file 1 **Table 1**. **Expected values for pedometer-determined physical activity in healthy older adults. **[2-29]. Click here for file
