# Supplementary material for: Correction: Expected values for pedometer-determined physical activity in older populations
Source: Int J Behav Nutr Phys Act. 2009 Oct 9;6:65. doi: 10.1186/1479-5868-6-65 (PMC2765970; doi:10.1186/1479-5868-6-65)
Supplement: Additional file 1 — Table 1. Expected values for pedometer-determined physical activity in healthy older adults. [2-29]. [file 1479-5868-6-65-S1.DOC]

Table 1. Expected values for pedometer-determined physical activity in healthy older adults

| **Study** | **Sample** | **Age (years)** | **Pedometer** | **Monitoring frame**  **(sealed/unsealed)** | **Mean steps/day** | **SD*** |
| --- | --- | --- | --- | --- | --- | --- |
| Moreau et al.[2]  (United States) | 24 post menopausal females | 54±1 | Yamax SW-200, Yamax, Inc., Tokyo, Japan | 1-2 weeks  (unsealed) | Exercise 5400^a^  Control 7200 | SE 500  SE 700 |
| Zhang et al. [3]  (Japan) | 141 males, 135 females | range 50-69 | Yamasa, EC-500, Japan | 3 days  (unsealed) | Males 8053  Females 6603 | 3695  2938 |
| King et al. [4]  (United States) | 149 females | mean 74.2±4.2 | Yamax DigiWalker | 7 days  (unsealed) | median 5285 | not reported |
| Fukukawa et al. [5]  (Japan) | 183 male, 131 female Japanese community-dwelling adults | range 65-79 | Select II, Suzuken Co., Nagoya, Japan | 7 days  (not indicated) | 5281^b^ | 2214 |
| Yamakawa et al.[6]  (United States) | 35 males, 47 females | 65.6±7.0  range 55-79 | Digi-Walker SW-701 | 7 days  (unsealed) | Total sample 5481.1  Normal weight 7385  Obese 3325 | 3628.9  3680  2346 |
| Jensen et al. [7]  (United States) | 26 females | 64.1±4.8  range 60-75 | Accusplit, San Jose, CA | not reported  (unsealed) | 4027 | 2515 |

| Tudor-Locke et al. [8]  (United States) | 45 older adults part of a larger survey 36% male, 64% female | 65+ | Yamax Model SW-200, Yamax Corportion, Tokyo, Japan | 7 days  (unsealed) | 3766 | 2805 |
| --- | --- | --- | --- | --- | --- | --- |
| Croteau et al. [9]  (United States) | 10 males, 66 females | range 60-90 | Accusplit AX120 step counter, Accusplit, San Jose, California | 7 days  (sealed) | Total sample 4041  <65 years 5314  65-69 years 5085  70-74 years 3810  75-79 years 3653  80-84 years 2688  85+ years 2015 | 2824  2316  4794  2444  1388  983  1538 |

| King et al. [10]  (United States) | 158 postmenopausal females | 57.3±3.0  range 52-62 | Yamax Accusplit (Accusplit Inc., San Jose, CA) | 7 days  (unsealed) | Caucasian 6840.3  African American 4526.3  < bachelor’s degree 6363.4  ≥ bachelor’s degree  7303.1  Work or volunteer  7417.0  Do not work or volunteer  6491.8  Married/living with partner  6825.6  Divorced/widowed/  separate/single  5816.0  Current smoker 5805.5  Non current smoker  6873.9  Summer 6240.1  Spring 6752.3  Summer 7195.6  Fall 6420.1 | not reported |
| --- | --- | --- | --- | --- | --- | --- |
| Wyatt et al. [11]  (United States) | 344 older adults part of a larger survey 49.3% male, 50.7% female | 50+ | Yamax Model SW-200, Yamasa Corportion, Tokyo, Japan | 4 days  (unsealed) | 50-59 years 6657.1  60+ years 5022.2 | SE 313.3  SE 315.9 |
| Aree-Ue et al. [12]  (Thailand) | 42 males, 1 female | 69.26±5.07  range 60-80 | FreeStyle PacerPro (FreeStyle, 2002) | 7 days  (unsealed) | 2597^c^ | 1565 |
| Krumm et al. [13]  (United States) | 93 postmenopausal females | mean 60.9±5.8  range 50-75 | Digi-Walker SW-200, New Lifestyles, Inc., Lees Summit, MO) | 14 days  (unsealed) | 6813 | 2955 |
| Shimizu et al.[14]  (Japan) | 114 male, 170 females, healthy elderly | mean 71.0±0.3  range 65-86 | Kenz Lifecorder, Suzuken Co. Ltd., Nagoya, Japan | 14 days  (unsealed, but participants asked not to look at it) | Total sample 6216  Males 6507  Females 6010 | SE 168  SE 295  SE 200 |
| Yoshiuchi et al.[15]  (Japan) | 83 males, 101 females | 65-85 | Modified Kenz Lifecorder, Suzuken Co. Ltd., Nagoya, Japan | 1 year  (not indicated) | 6635 | 2750 |
| Cavanaugh et al.[16]  (United States) | 7 males, 21 females | 83.7±2.3 | StepWatch 3 Activity Monitor (SAM; Cyma Corporation, Mountlake Terrace, WA | 6 days  (instrument does not display data) | 9981.7 | SE 552.8 |
| Swartz et al.[17]  (United States) | 63 males, 151 females free of disease or any condition that may affect PA levels | mean 72.3±8.9  range 55-94 | Yamax SW-200 Yamax Corp., Tokyo, Japan | 7 days  (sealed) | Total sample 4424  White 5036  Non-white 3671 | 2917  3106  2478 |
| Sarkisian et al.[18] (United States) | 5 male, 41 female | mean 77 | Digiwalker Yamax DW-500, New Lifestyles, Inc., Kansas City, MO | 7 days  (sealed) | 3536 ^c^ | 2281 |
| Marshall [19]  (Australia) | 38 males, 65 females | minimum 50 years | Yamax SW-700 Yamasa, Corp., Tokyo, Japan | 7 days  (unsealed first week, sealed second week) | No pedometer feedback 8360 ^a,c^  Pedometer feedback 8768 | 3294  3705 |
| Kubo [20]  (Japan) | 15 males | 69.7±4.5  range 62-77 | FB-714, TANITA, Tokyo, Japan | 2 weeks  (unsealed) | 7025 | 2492 |

| Strath et al. [21]  (United States) | 150 older adults (sex breakdown not reported) | 72.4±9.1  range 55-87 | SW-200, Yamax Corp., Tokyo, Japan | 7 days  (sealed) | Total sample 3912  White 4556  Non-white 3589  Hispanic 3830  Asian/Hmong 3832  American Indian 3023 | 2757  3305  2392  2988  1961  1563 |
| --- | --- | --- | --- | --- | --- | --- |
| Rowe et al. [22]  (United States) | 29 males, 60 females | 74.0±9.5 | Yamax SW-200, Yamax Corp., Tokyo, Japan | 7 days  (unsealed) | 4728.4 | 3640.5 |
| Parker et al. [23]  (United States) | 28 males, 56 females | 71.3±8.4  range 55-87 | Yamax SW-200, Yamax, Corp., Tokyo, Japan | 7 days  (unsealed) | 5233 | 2982 |
| Tudor-Locke et al. [24]  (Australia) | 148 males and 171 females | 50+ | Yamax SW-200-024 | 7 days  (unsealed) | males 50-59.9 yrs 7833  females 50-59.9 yrs 8749  males 60+ yrs 7982  females 60+ yrs 7139 | 2908  3702  3261  2780 |
| Tudor-Locke et al. [25]  (secondary analysis of data originally collected from different countries) | 379 males  923 females | 50+ | Yamax (Yamax Corporation, Tokyo, Japan) | minimum 3 days  (not indicated) | normal weight males  51-88 years  8938  overweight/obese males 51-88 years  6924  normal weight females 50-59.9 years  8289  overweight/obese females 50-59.9 years  6580  normal weight females 60-94 years  7021  overweight/obese females 60-94 years  5362 | 3451  3318  3670  3324  3362  2801 |
| Woolf et al. [26]  (United States) | 47 females | 68.4±4.9 | Digi-Walker SW-200, Yamax Corportion, Tokyo, Japan | 7 days  (unsealed) | 8088 | 2941 |
| Opdenacker et al. [27] (Belgium) | 93 males, 87 females | mean 67 | Yamax Digwalker SW-200, Yamax Corporation, Japan | 5 days  (not indicated) | Structured 7390 ^a^  Lifestyle 6765  Control 7720 | SE 400  SE 397  SE 392 |
| Payn et al. [28] (United States) | 29 older adults (sex breakdown not reported) | ≥60 | Yamax Digi Walker SW-200, Yamax USA, Inc.,  San Antonia, TX | 7 days  (unsealed) | 5143 | 2459 |
| Fitzpatrick et al. [29] (United States) | 95 males, 497 females attending senior centers | 75±8 | Accusplit, San Jose, CA | 7 days  (unsealed) | 2895 | 2170 |

*SD unless indicated SE;  ^a^groups otherwise equivalent at baseline; ^b^baseline data reported from a longitudinal study; ^c^steps/day values imputed from reported steps/week divided by 7
